# Renoprotective effects of empagliflozin are linked to activation of the tubuloglomerular feedback mechanism and blunting of the complement system

**DOI:** 10.1152/ajpcell.00528.2022

**Published:** 2023-02-13

**Authors:** Xin Chen, Denis Delić, Yaochen Cao, Linghong Shen, Qin Shao, Zheyu Zhang, Hongwei Wu, Ahmed A. Hasan, Christoph Reichetzeder, Mohamed M. S. Gaballa, Bernhard K. Krämer, Thomas Klein, Lianghong Yin, Ben He, Stanislao Morgera, Berthold Hocher

**Affiliations:** ^1^Department of Nephrology, Charité—Universitätsmedizin Berlin, Campus Mitte, Berlin, Germany; ^2^Fifth Department of Medicine (Nephrology/Endocrinology/Rheumatology/Pneumology), University Medical Centre Mannheim, University of Heidelberg, Heidelberg, Germany; ^3^The First Clinical Medical College of Jinan University, The First Affiliated Hospital of Jinan University, Guangzhou, People’s Republic of China; ^4^Department of Translational Medicine & Clinical Pharmacology, Boehringer Ingelheim Pharma GmbH & Co. KG, Biberach, Germany; ^5^Department of Cardiology, Shanghai Chest Hospital, Shanghai Jiao Tong University School of Medicine, Shanghai, People’s Republic of China; ^6^Institute of Pharmacy, Freie Universität Berlin, Berlin, Germany; ^7^Department of Pathology, HMU — Health and Medical University, Potsdam, Germany; ^8^Faculty of Veterinary Medicine, Benha University, Toukh, Egypt; ^9^European Center for Angioscience, Medical Faculty Mannheim, University of Heidelberg, Heidelberg, Germany; ^10^Department of Cardiometabolic Diseases Research, Boehringer Ingelheim Pharma GmbH & Co. KG, Biberach, Germany; ^11^Reproductive and Genetic Hospital of CITIC-Xiangya, Changsha, People’s Republic of China; ^12^IMD Institut für Medizinische Diagnostik Berlin-Potsdam GbR, Berlin, Germany

**Keywords:** complement system, nondiabetic chronic kidney disease, sodium-glucose cotransporter 2 inhibitor, tubuloglomerular feedback

## Abstract

The mechanisms of nephroprotection in nondiabetic chronic kidney disease (CKD) models by sodium-glucose cotransporter 2 (SGLT2) inhibitors are not well defined. Five groups were established: sham-operated rats, placebo-treated rats with 5/6 nephrectomy (5/6Nx), 5/6Nx + telmisartan (5 mg/kg/day), 5/6Nx + empagliflozin (3 mg/kg/day), and 5/6Nx + empagliflozin (15 mg/kg/day). Treatment duration was 95 days. Empagliflozin showed a dose-dependent beneficial effect on the change from baseline of creatinine clearance (Ccr). The urinary albumin-to-creatinine ratio likewise improved in a dose-dependent manner. Both dosages of empagliflozin improved morphological kidney damage parameters such as renal interstitial fibrosis and glomerulosclerosis. 5/6 nephrectomy led to a substantial reduction of urinary adenosine excretion, a surrogate parameter of the tubuloglomerular feedback (TGF) mechanism. Empagliflozin caused a dose-dependent increase in urinary adenosine excretion. The urinary adenosine excretion was negatively correlated with renal interstitial fibrosis and positively correlated with Ccr. Immunofluorescence analysis revealed that empagliflozin had no effect on CD8^+^ and CD4^+^ T cells as well as on CD68^+^ cells (macrophages). To further explore potential mechanisms, a nonhypothesis-driven approach was used. RNA sequencing followed by quantitative real-time polymerase chain reaction revealed that complement component 1Q subcomponent A chain (*C1QA*) as well as complement component 1Q subcomponent C chain (*C1QC*) gene expression were upregulated in the placebo-treated 5/6Nx rats and this upregulation was blunted by treatment with empagliflozin. In conclusion, empagliflozin-mediated nephroprotection in nondiabetic CKD is due to a dose-dependent activation of the TGF as well as empagliflozin-mediated effects on the complement system.

## Graphical abstract

**Figure:**
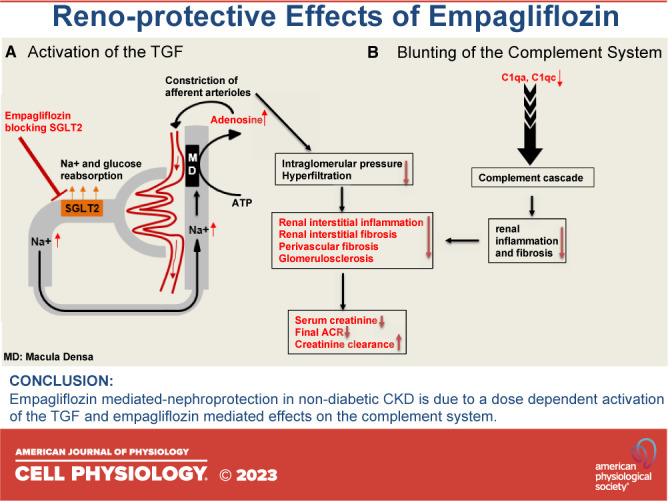

## INTRODUCTION

Chronic kidney disease (CKD) arises from a number of heterogeneous disease pathways that irreversibly alter the function and structure of the kidney (1). According to worldwide estimates, there are nearly 700 million prevalent patients with chronic kidney disease(2). In addition to primary kidney disease, common chronic diseases such as hypertension and diabetes harm the kidneys and hence contribute to the burden of CKD (3). Although the causes of CKD are diverse, they all culminate in progressive glomerulosclerosis, tubular atrophy, and interstitial fibrosis (4). No pharmacological treatment approach has been found to be effective in halting or reversing the progressive progression of CKD in humans, and renal replacement therapy (dialysis or kidney transplantation) remains the main burden from a health economic point of view very costly treatment for the end-stage kidney disease.

Sodium-glucose cotransporter 2 (SGLT2) inhibitors are antidiabetic drugs that act primarily on SGLT2 expressed in the proximal tubule of the kidney, thereby reducing renal reabsorption of glucose and also sodium (5). These compounds have received special attention because several large-scale clinical trials have shown nephroprotective effects and a reduction in the incidence of cardiovascular events (6–9). However, most of these clinical trials were conducted in patients with diabetic nephropathy, and it is unclear to what extent the nephroprotective effect of SGLT2 inhibitors in patients results from a reduction in blood glucose. In fact, in a large clinical study with 4,304 nondiabetic patients with CKD, SGLT2 inhibitors had a beneficial effect on glomerular filtration rate (GFR) and reduced mortality (10). Another clinical study with 6,609 patients with CKD showed that empagliflozin therapy led to a lower risk of progression of kidney disease (11). This fits very well with earlier post hoc analyses of clinical trials showing that the nephroprotective effect of SGLT2 inhibitors were independent of hemoglobin A1c (HbA1c) and blood glucose levels (9). These evidences suggest that SGLT2 inhibitors are likely to have other beneficial effects independent of lowering blood glucose, but the underlying mechanisms of nephroprotective effects remain unclear. Current hypotheses mainly involve effects on inflammation(12), hypoxia (13), sympathetic nervous system (14), tubuloglomerular feedback (TGF) (15), and metabolism (16, 17). Nevertheless, there are limitations regarding these experiments, such as the fact that most animal experiments were done just in diabetic models, that drug treatment was rather short hence statements on long-term efficacy and safety could not be made. Finally, mainly hypothesis-driven approaches to prove efficacy on predefined pathways were usually used. Yet unknown mechanisms thus could not be identified.

Accordingly, the aim of this study was to investigate the effects of empagliflozin treatment in nondiabetic CKD rats (5/6 nephrectomy rat model) for 95 days on renal function and structure, as well as on body weight, and blood pressure. Two doses of empagliflozin (3 mg/kg/day and 15 mg/kg/day) were used to investigate potential dose dependency and telmisartan as the gold standard for nephroprotective effects were used (18–21). To further explore the potential underlying mechanisms, RNA sequencing was used to search for differentially expressed genes due to SGLT2 treatment. The resulting findings were then validated using quantitative real-time polymerase chain reaction (qRT-PCR).

## METHODOLOGY

### Experimental Animals and Protocol

Animal experiments were approved by the Animal Care and Use Committee of Jinan University, Guangzhou, PR China (IACUC-20190830-02). All animals were cared for in accordance with the Guide for the Care and Use of Laboratory Animals published by the US National Institute of Health (NIH Publication No. 85-23, revised 1996).

Male Wistar rats (15 animals per group, except for 10 animals in sham group), aged 7 wk, were purchased from Vital River Laboratory Animal Technology Co., Ltd (Beijing). The rats were kept at temperature-controlled conditions (22°C–25°C), humidity of 55 ± 5%, under 12 h light/dark cycles and were allowed access to standard rat chow and water ad libitum. The general condition of each animal was monitored daily. After 7 days of acclimatization, rats were randomly assigned into five different groups, sham operation + placebo; 5/6Nx + placebo; 5/6Nx + telmisartan (5 mg/kg/day); 5/6Nx + empagliflozin (3 mg/kg/day); 5/6Nx + empagliflozin (15 mg/kg/day) and were treated with medication for 95 consecutive days by gavage immediately after surgery until the end of the study (24 h before euthanasia). Doses of telmisartan (22, 23) and empagliflozin (24–26) were selected based on prior studies. The five-sixths nephrectomy (5/6Nx) operation under anesthesia with 2,2,2-tribromethanol (500 mg/kg ip) was performed as follows: uninephrectomy of the right kidney (Uni-Nx) at *week 1* followed by amputation of the poles of the left kidney at *week 3* (Supplemental Figs. S1 and S2). Sham operations were conducted at the same time points. The rats were weighed every fortnight. Blood pressure (BP) measurements using the tail-cuff method (BP-2000 Blood Pressure Analysis System, model BP-2000-RP-4, Visitech Systems), metabolic cages, and blood sampling were performed at *weeks 0*, *7*, and *18* (Supplemental Figs. S1and S2). Urine was collected by placing the rats in metabolic cages (MC) for 24 h, and the 24 h urine volume was recorded. After centrifugation at 12,000 rpm for 10 min at 4°C, the urine supernatant was stored at −80°C for further analysis. Except for blood samples at *week 18*, that were taken from the abdominal aorta under anesthesia with 2,2,2-tribromethanol (500 mg/kg ip) and placed in blood collection tubes (BD Vacutainer SST II Advance Serum Tube with Separating Gel and Clotting Activator) during euthanasia process, blood samples at *weeks 0* and *7* were taken from retro-orbital venous plexus under anesthesia with isoflurane via chamber induction (3%), and the supernatants of blood samples were stored at −80°C after centrifugation at 3,000 rpm for 10 min at 4°C. The rats were euthanized at *week 18*. Upon euthanasia, blood samples were taken and kidneys were harvested. The kidneys were weighed and cut longitudinally into two halves: one half was fixed in 4% paraformaldehyde for further histological analysis and the other half was stored at −80°C with RNA tissue protection reagent (No.76106, Qiagen, Germany) for later analyzes.

### Serum and Urine Analyses

Levels of serum creatinine and urinary creatinine were analyzed with automatic biochemical analyzer (Siemens biochemical analyzer and its Leadman reagent, Siemens, Germany). Urinary albumin levels were determined quantitatively using enzyme-linked immunosorbent assay kit (ab235642, Abcam, Cambridge, UK). Determination of urinary adenosine excretion (urine adenosine/urine creatinine) was performed as described previously (27). Plasma levels of empagliflozin were determined by high-performance liquid chromatography (HPLC), as described previously (28, 29). The urinary albumin-to-creatinine ratio (ACR) and creatinine clearance (Ccr) were calculated by formulas as follows: ACR = urinary albumin/urine creatinine; Ccr = [urinary creatinine (μmol/L) × urinary flow (mL/min)]/serum creatinine (μmol/L).

### Histology

Renal tissues were embedded in paraffin after fixation in 4% paraformaldehyde for 48 h. The embedded renal tissues were cut into 2-µm-thick sections, then stained with periodic acid-Schiff staining (PAS), Sirius Red staining, periodic Schiff-methenamine staining (PASM), and Masson’s trichrome staining (Masson) to evaluate glomerulosclerosis index, glomerular size, number of glomerular cells, renal interstitial fibrosis, and perivascular fibrosis index. All slides were scanned in full at ×400 magnification, and the images were viewed and analyzed by iViewer 6.3.6 (Unic Technologies, Inc., PR China). Glomerulosclerosis index was evaluated on PAS-stained slides by rating the percentage of the periodic acid-Schiff-positive areas within the glomerulus using a subjective, semiquantitative scoring system (*grades I–IV*) performed by two investigators who were blinded to the study groups. Analysis of perivascular fibrosis index was done by two blinded independent investigators using a semiquantitative grading score (*grades I–IV*) on Masson’s trichrome-stained slides as follow: *I* = weak, *II* = moderate, *III* = severe, and *IV* = very severe fibrosis per section. Images for the measurement of renal interstitial fibrosis were intercepted from full-scan images of Sirius Red staining at ×400 magnification, and the images assessed covered more than 80% of the entire slide. Subsequently, the percentage of fibrosis on these images was measured by the ImageJ threshold method (National Institutes of Health), as previously described (30). Glomerular size and number of glomerular cells were assessed by analyzing 50 glomeruli of each PASM-stained slide using iViewer 6.3.6 (Unic Technologies, Inc., PR China). For all of the aforementioned analyses, at least 80% field per slide was assessed by two blinded independent investigators, and the mean of the two scoring results was used.

### Immunofluorescence Staining

For immunofluorescence, 4-µm-thick slides were used. Frozen sections were prepared and immunofluorescence analysis was conducted using primary antibodies as follows: anti‐CD68 (GB113109, Servicebio, PR China, dilution 1:2,000), anti‐CD8 (GB11068, Servicebio, PR China, dilution 1:500), and anti‐CD4 (GB11064-1, Servicebio, PR China, dilution 1:500) overnight at 4°C. Then, the tissues and cells were washed and incubated with secondary antibody (anti‐CD68: GB22303, Servicebio, PR China, dilution 1:500; anti‐CD8 and anti‐CD4: GB21303, Servicebio, PR China, dilution 1:300). After staining the nuclei with 4′,6-diamidino-2-phenylindole (DAPI), the tissues and cells were visualized. For each section, 12 randomized high-power fields were examined using a fluorescence microscope at ×200 magnification. Cytotoxic T cells (CD8^+^ cells/total cells) and helper T cells (CD4^+^ cells/total cells) were analyzed by AIpathwell (Servicebio, PR China). The relative fluorescence unit (RFU) of CD68 was assessed by ImageJ software (National Institutes of Health). When evaluating RFUs, nonconforming images are excluded: *1*) areas with an unspecific staining pattern due to the edge effect; *2*) damaged or obscured tissue areas; *3*) areas outside our focus areas; *4*) an area with indistinct nuclei staining; and *5*) areas include irrelevant tissue features like large blood vessels or large spaces.

### RNA Sequencing and Quantitative Real-Time Polymerase Chain Reaction (qRT-PCR)

Six renal tissue samples from animals with a serum creatinine level close to the median serum creatinine level at study end in each group were selected for RNA sequencing analysis using a HiSeq 4000 platform device (Illumina, Inc; San Diego, CA). Library preparation, sequencing, and data analysis of messenger RNA (mRNA) were performed, as described previously (23). The 5/6Nx + placebo (PBO) group was used as a control for group comparison. Genes with an absolute value of log2(fold-change) > 1 and a *P* value <0.05 were considered as differentially expressed genes (DEGs) (Supplemental Figs. S1–S3). Results were corrected for multiple testing. The selected differentially expressed genes were ranked from lowest to highest *P* value, and the top six functional genes that were simultaneously regulated in the control (5/6Nx + PBO and 5/6Nx + TELM) and the intervention (5/6Nx + 3 mg EMPA and 5/6Nx + 15 mg EMPA) groups were validated by qRT-PCR. Using these selection criteria, top differentially expressed genes (DEGs) were retested by qRT-PCR (SDS7900HT; Thermo Fisher Scientific, Inc., Waltham, MA). Eventually, *C1QA* (complement component 1, Q subcomponent, A chain), *C1QC* (complement component 1, Q subcomponent, C chain), *FMOD* (fibromodulin), *REN* (renin), *FMO2* (flavin-containing dimethylaniline monooxygenase 2), and *RPL36* (ribosomal protein L36) were verified by qRT-PCR. These six genes were also labeled in the Volcano plot (Supplemental Figs. S1–S3).

### Statistical Analysis

Data were presented as the means ± SE. Statistical analysis was performed using GraphPad Prism 6 software (GraphPad, La Jolla, CA). ROUT (Q = 1%) in GraphPad Prism 6 software was used to identify outliers. Only two outliers were excluded (one from the 5/6Nx + TELM group for creatinine clearance and the other from the +PBO group for glomerular size). For analysis of serum creatinine over the course of the study, two-way analysis of variance (two-way ANOVA) followed by the Bonferroni post hoc test was performed. In all other cases, one-way ANOVA analysis followed by the Bonferroni post hoc test was applied for comparison of normally distributed data, and the Kruskal-Wallis test followed by Dunn’s post hoc test was used for nonnormally distributed data. In all cases, differences were regarded as statistically significant if *P* < 0.05.

### Materials

Empagliflozin (EMPA) and telmisartan (TELM) were manufactured and supplied by Boehringer Ingelheim Pharma GmbH & Co. KG (Biberach an der Riss, Germany). Both compounds were dissolved in 0.5% wt/vol hydroxypropyl methylcellulose in water and were administered orally by gavage. Control rats received 0.5% wt/vol hydroxypropyl methylcellulose in water.

## RESULTS

### Physiologic and Biochemical Data

**Figure 1.**
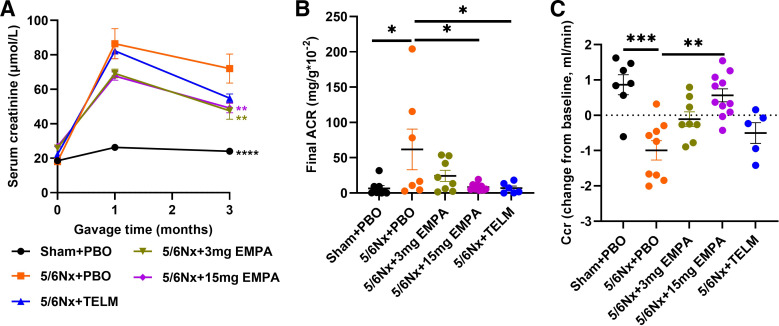
Effect on renal function compared with 5/6Nx + PBO group. *A*: concentrations of serum creatinine over the course of the study: Sham + PBO (*n* = 7), 5/6Nx + 3 mg EMPA (*n* = 8) and 5/6Nx + 15 mg EMPA (*n* = 11) were lower than 5/6Nx + PBO (*n* = 9). *B*: final ACR: Sham + PBO (*n* = 8), 5/6Nx + 15 mg EMPA (*n* = 9) and 5/6Nx + TELM (*n* = 6) were lower than 5/6Nx + PBO (*n* = 7). *C*: Ccr (change from baseline): Sham + PBO (*n* = 7) and 5/6Nx + 15 mg EMPA (*n* = 11) were higher than 5/6Nx + PBO (*n* = 9). Values displayed are means ± SE. *P < 0.05; **P < 0.01; ***P < 0.001; ****P < 0.0001, significantly different from 5/6Nx + PBO. EMPA, empagliflozin; 5/6Nx, 5/6 nephrectomized rat model; PBO, placebo; Sham, sham operation; TELM, telmisartan. ACR, urinary albumin-to-creatinine ratio; Ccr, creatinine clearance.

The physiological and biochemical data of the animals are shown in Table 1. There was no statistical difference in blood pressure between all groups neither at study entry nor at study end (data not shown). Empagliflozin demonstrated a dose-dependent beneficial effect on the change from baseline of Ccr. The lower dose of empagliflozin showed only a trend toward improvement compared with placebo. The overall picture, however, indicates a dose dependency of the empagliflozin effects on Ccr. The urinary albumin-to-creatinine ratio was determined only at the end of the experiment. Here, too, a dose-dependent beneficial effect of empagliflozin can be seen. The nephroprotective effects of empagliflozin at the higher dose were superior to treatment effects of a standard dose of the angiotensin II receptor blocker telmisartan, the current gold standard for the treatment of CKD (Table 1 and Fig. 1).

**Table 1.** Changes of animal characteristics from baseline

Parameters	Sham + PBO (*n* = 7)	5/6Nx + PBO (*n* = 9)	5/6Nx + 3 mg EMPA (*n* = 9)	5/6Nx + 15 mg EMPA (*n* = 11)	5/6Nx + TELM (*n* = 7)
Body weight (change from baseline, g)	208.57 ± 20.75*	149.11 ± 11.60	149.56 ± 17.53	146.36 ± 7.23	139.71 ± 15.05
Rel. kidney weight (10^−2^)	0.41 ± 0.02	0.51 ± 0.06	0.55 ± 0.05	0.61 ± 0.03	0.41 ± 0.03
Serum creatinine (change from baseline, μmol/L)	5.71 ± 0.81****	53.78 ± 8.85	23.00 ± 4.94***	22.91 ± 2.29***	37.14 ± 3.46
Final ACR (mg/g × 10^−2^)	6.66 ± 3.83*	61.75 ± 28.79	24.28 ± 7.85	8.48 ± 1.70*	6.88 ± 3.10*
Ccr (change from baseline, mL/min)	0.86 ± 0.28***	−0.99 ± 0.27	−0.11 ± 0.21	0.56 ± 0.18**	−0.50 ± 0.29
*T*_24_ (empagliflozin, nM)	–	–	–	23.20 ± 6.50	–

Body weight (change from baseline) = final body weight – baseline body weight; rel. kidney weight (relative kidney weight) = kidney weight/final body weight; ACR (urinary albumin-to-creatinine ratio) = urinary albumin/urine creatinine; Ccr (creatinine clearance) = [urinary creatinine (μmol/L) × urinary flow (mL/min)]/serum creatinine (μmol/L); Ccr (change from baseline) = final Ccr – baseline Ccr; *T*_24_ (empagliflozin, nM): plasma concentrations of empagliflozin after 24 h of gavage. – indicates below the minimum detection value. Values displayed are means ± SE. *P < 0.05; **P < 0.01; ***P < 0.001; ****P < 0.0001, significantly different from 5/6Nx + PBO. EMPA, empagliflozin; 5/6Nx, 5/6 nephrectomized rat model; PBO, placebo; Sham, sham operation; TELM, telmisartan.

### Kidney Morphology

**Figure 2.**
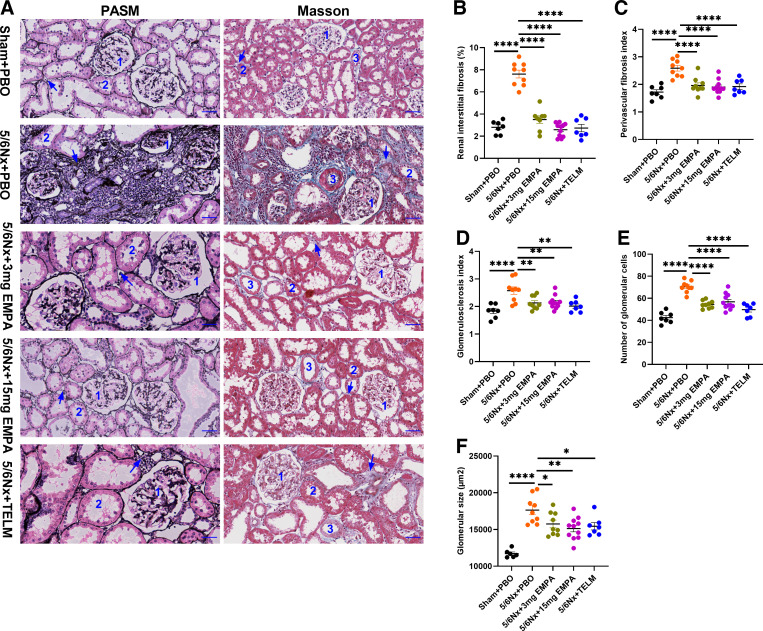
Histopathological analysis of kidney compared with 5/6Nx + PBO group. *A*: representative micrographs of PASM and Masson staining of kidney sections from each group (×400, scale bar = 50 μm); *Label 1*: glomerulus; *Label 2*: renal tubules; *Label 3*: vascular; arrow: renal interstitial fibrosis. Renal interstitial fibrosis (%; *B*); perivascular fibrosis index (*C*); glomerulosclerosis index (*D*); number of glomerular cells (*E*); glomerular size (µm^2^; *F*). All these indicators were lower in the other groups [Sham + PBO (*n* = 7), 5/6Nx + TELM (*n* = 7), 5/6Nx + 3 mg EMPA (*n* = 9) and 5/6Nx + 15 mg EMPA (*n* = 11)] than in the 5/6Nx + PBO (*n* = 9) group. Values displayed are means ± SE. *P < 0.05; **P < 0.01; ***P < 0.001; ****P < 0.0001, significantly different from 5/6Nx + PBO. EMPA, empagliflozin; 5/6Nx, 5/6 nephrectomized rat model; PASM, periodic Schiff-methenamine staining; PBO, placebo; Sham, sham operation; TELM, telmisartan.

All histological parameters of kidney damage (renal interstitial fibrosis, perivascular fibrosis index, glomerulosclerosis index, number of glomerular cells and glomerular size) were clearly pronounced after 5/6Nx compared with the sham-operated rats (Fig. 2, *A*–*F*). Both the low and high doses of empagliflozin improved all these morphological kidney damage parameters (Fig. 2, *A*–*F*). Treatment with both dosages of empagliflozin led to an almost complete regression of the pathological changes of renal interstitial fibrosis, perivascular fibrosis index, glomerulosclerosis index, and number of glomerular cells (Fig. 2, *A*–*E* and Supplemental Fig. S2). The effect on glomerular size was significant, but less pronounced. Here, as with the functional parameters, see Fig. 1, some dose dependency was suggested (Fig. 2*F*). The angiotensin II receptor blocker telmisartan improved all analyzed kidney damage parameters significantly (Fig. 2, *A*–*F*).

### Urinary Adenosine

**Figure 3.**
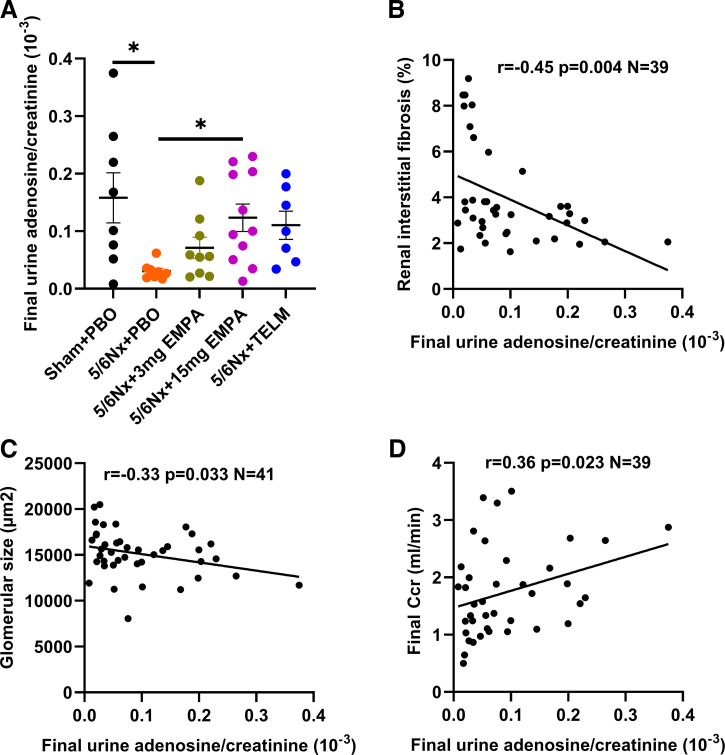
*A*: comparison of final urine adenosine/urine creatinine between each treatment group and 5/6Nx + PBO. Values displayed are means ± SE. *P < 0.05, significantly different from 5/6Nx + PBO. Inverse correlation between urinary adenosine/creatinine ratio at study end with renal interstitial fibrosis (*r* = −0.45, *P* = 0.004, *N* = 39; *B*) and glomerular size (*r* = −0.33 *P* = 0.033 *N* = 41; *C*). Positive correlation between urinary adenosine/creatinine ratio at study end with GFR (*r* = 0.36, *P* = 0.023, *N* = 39; *D*). EMPA, empagliflozin; 5/6Nx, 5/6 nephrectomized rat model; PBO, placebo; Sham, sham operation; TELM, telmisartan. Ccr, creatinine clearance.

The 5/6 nephrectomy led to a drastic reduction in urinary adenosine excretion. Empagliflozin induced a dose-dependent increase in urinary adenosine excretion. The higher dose group of empagliflozin was significantly different from placebo-treated rats with 5/6 nephrectomy in terms of urinary adenosine excretion (Fig. 3*A*). Telmisartan treatment showed a trend toward increasing the urine adenosine to urine creatinine ratio (*P* = 0.057, Fig. 3*A*). Urinary adenosine to creatinine ratio at study end was negatively correlated with renal interstitial fibrosis (Fig. 3*B*) and glomerular size (Fig. 3*C*) and positively correlated with Ccr at study end (Fig. 3*D*).

### Kidney Immunofluorescence

**Figure 4.**
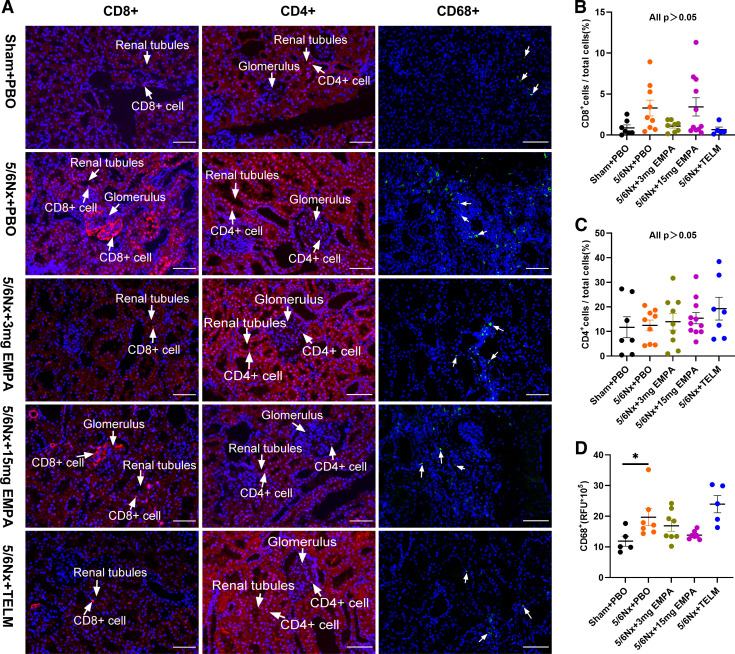
Immunofluorescence staining analysis of kidney. *A*: representative micrographs of kidney sections from each treatment group (×200, scale bar = 100 μm). *B*: cytotoxic T cells (CD8^+^ cells/total cells, %): no statistical difference between groups (Sham + PBO: *n* = 7, 5/6Nx + TELM: *n* = 7, 5/6Nx + 3 mg EMPA: *n* = 9; 5/6Nx + 15 mg EMPA: *n* = 11; 5/6Nx + PBO: *n* = 9). *C*: helper T cells (CD4^+^ cells/total cells, %): no statistical difference between groups (Sham + PBO: *n* = 7, 5/6Nx + TELM: *n* = 7, 5/6Nx + 3 mg EMPA: *n* = 9; 5/6Nx + 15 mg EMPA: *n* = 11; 5/6Nx + PBO: *n* = 9). *D*: macrophages (CD68+, RFU): 5/6Nx + PBO was higher than Sham + PBO group (Sham + PBO: *n* = 5, 5/6Nx + TELM: *n* = 5, 5/6Nx + 3 mg EMPA: *n* = 8; 5/6Nx + 15 mg EMPA: *n* = 7; 5/6Nx + PBO: *n* = 7). The arrows point to CD68+ cells. Values displayed are means ± SE. *P < 0.05, significantly different from 5/6Nx + PBO. EMPA, empagliflozin; 5/6Nx, 5/6 nephrectomized rat model; PBO, placebo; RFU, relative fluorescence unit; Sham, sham operation; TELM, telmisartan.

Some studies (31, 32), including our own recently published study (33), suggest that SGLT2 inhibitor ameliorates renal fibrosis in CKD rats by affecting macrophage and inflammatory cells. We therefore detect the number of CD4^+^ and CD8^+^ cells, as well as the CD68+ cells in kidney tissues. However, there were no statistical differences between 5/6Nx + PBO and the other groups with regard to kidney tissue density of cytotoxic T cells (CD8^+^ cells/total cells) and T helper cells (CD4^+^ cells/total cells) (Fig. 4, *A*–*C*). The tissue density of CD68+ cells (macrophages) was increased in 5/6Nx + PBO group (*P* = 0.047) versus sham-operated animal. However, neither empagliflozin nor telmisartan treatment had an effect on the tissue density of macrophages after 5/6 nephrectomy (Fig. 4*D*). Taken together, neither empagliflozin nor telmisartan had an effect on CD8^+^ T cells and CD4^+^ T cells, and also on CD68+ cells (macrophages).

### RNA Sequencing and Quantitative Real-Time Polymerase Chain Reaction (qRT-PCR) of Kidney Samples

**Figure 5.**
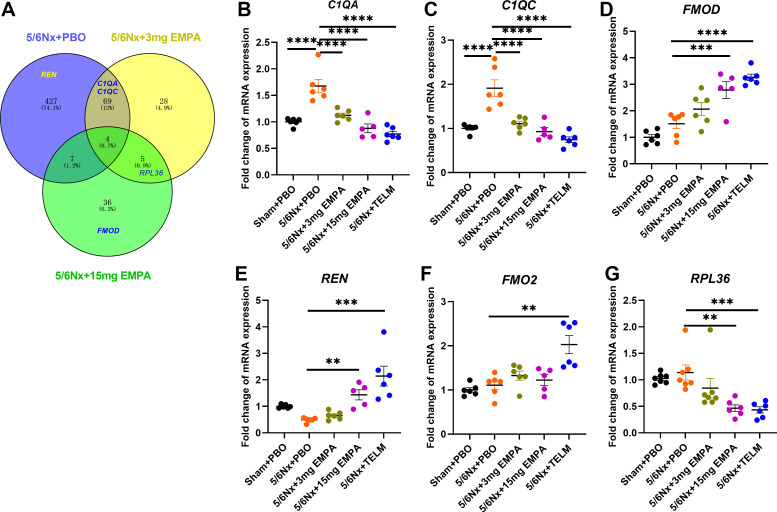
Gene expression in the kidney identified by qRT-PCR. *A*: Venn diagram of the differentially expressed genes from RNA-seq transcriptome for 5/6Nx + PBO, 5/6Nx + 3 mg EMPA and 5/6Nx + 15 mg EMPA groups. The circles represent the total number of statistically significant different genes [*P* value < 0.05 and an absolute value of log2(fold-change) > 1] in 5/6 Nx + 3 mg EMPA and 5/6 Nx + 15 mg EMPA groups in comparison to 5/6 Nx + PBO in kidney according to RNA-seq transcriptome data. *B*: fold-change of *C1QA* expression (complement component 1, Q subcomponent, A chain). *C*: fold-change of *C1QC* expression (complement component 1, Q subcomponent, C chain). *D*: fold-change of *FMOD* expression (fibromodulin). *E*: fold-change of *REN* expression (renin). *F*: fold-change of *FMO2* expression (flavin-containing dimethylaniline monooxygenase 2). *G*: fold-change of *RPL36* expression (ribosomal protein L36). Values displayed are means ± SE. **P < 0.01; ***P < 0.001; ****P < 0.0001, significantly different from 5/6Nx + PBO. EMPA, empagliflozin; 5/6Nx, 5/6 nephrectomized rat model; PBO, placebo; qRT-PCR, quantitative reverse transcription polymerase chain reaction; Sham, sham operation; TELM, telmisartan.

Venn diagram between 5/6Nx + 3 mg EMPA, 5/6Nx + 15 mg EMPA and 5/6Nx + PBO groups showed 4 coregulated genes between these three groups, 73 coregulated genes between 5/6Nx + 3 mg EMPA and 5/6Nx + PBO groups, and 11 coregulated genes between 5/6Nx + 15 mg EMPA and 5/6Nx + PBO groups (Fig. 5*A*). Using the selection criteria (top-fold-change, detection in several comparisons, and known function, for details, see method section), RNA sequencing yielded six candidate genes for verification with qRT-PCR in all samples: complement component 1Q subcomponent A chain (*C1QA*); complement component 1Q subcomponent C chain (*C1QC*); fibromodulin (*FMOD*); renin (*REN*); flavin-containing dimethylaniline monooxygenase 2 (*FMO2*); and ribosomal protein L36 (*RPL36*) (Supplemental Figs. S1–S3 and Fig. 5). Only the *C1QA* and *C1QC* genes showed a model effect—means were upregulated after 5/6 nephrectomy—and responded to empagliflozin treatment (Fig. 5, *B* and *C*). Renin expression was not altered significantly by 5/6 nephrectomy but increased substantially after treatment with the angiotensin II receptor blocker telmisartan, a well-known effect of telmisartan (34, 35).

## DISCUSSION

This study provides evidence of a dose-dependent, glucose-independent nephroprotection by the SGLT2 inhibitor empagliflozin. These nephroprotective effects of the SGLT2 inhibition were accompanied by dose-dependent effects on a surrogate parameter of the TGF mechanism: urinary adenosine excretion. Besides that, whole genome RNA sequencing followed by qRT-PCR confirmation identified alterations in gene expression of key genes of the complement system in the kidney. Activation of the complement system has been shown to be a key mechanism of kidney injury.

Our data on the nephroprotective effects of SGLT2 inhibition by empagliflozin fits to previous findings in patients with diabetes (8–10) and also to findings in animal models of diabetic nephropathy (36–38) and even to findings in nondiabetic nephropathy models (39–43), However, there are also some studies showing no effect of SGLT2 inhibition in nondiabetic CKD models. Nishiyama et al. (44) found that SGLT2 inhibitor (TA-1887, 10 mg/kg/day) treatment of 5/6 nephrectomized rats for 10 wk had no beneficial effects on serum creatinine clearance, proteinuria and renal tissue structure. Gilbert and colleagues (45) showed that 0.5 mg/kg dapagliflozin twice/day for 12 wk had no effect on renal function or structure in 5/6 nephrectomized rats. Since different dosages were not tested in current studies, one might speculate that dosing in these studies or compound-related effects could account for these differences. The effects of different SGLT2 inhibitor compounds may also vary. Some current clinical studies showed that empagliflozin is more effective than dapagliflozin (46, 47). Besides that, the choice of the CKD model seems to matter. Tauber et al. (48) using an adenine-induced CKD model and Vervaet and colleagues (49) using a CKD model with unilateral nephrectomy on high-salt diets detected no beneficial response to SGLT2 inhibition. In clinical practice, however, SGLT2 inhibitors seem to decrease chronic kidney disease progression independently of the underlying type of initial kidney injury.

It was suggested that the nephroprotective effects of the SGLT2 inhibitor empagliflozin are most likely due to its effect on the tubuloglomerular feedback (TGF) mechanism (14, 50, 51). The TGF regulates glomerular blood flow and hence glomerular. This is a physiological counter-regulation that protects individual nephrons from hyperfiltration, increased intraglomerular pressure and sodium and fluid overload of the tubules. TGF is based on the osmosensory cells of the macula densa, which are part of the juxtaglomerular apparatus. They measure the concentration of sodium and chloride ions in the lumen of the distal tubule via the Na-K-2Cl cotransporter (52–54). Huge amounts of the filtered sodium in the glomerulus are reabsorbed in the S1 segment of the proximal tubule of the nephron by a transporter called sodium-glucose cotransporter 2 (SGLT2). Inhibition of SGLT2 in the S1 segment of the proximal tubule leads to increased sodium at the macula densa of the distal tubule, which causes constriction of the afferent arteriole and reduction of glomerular filtration pressure via paracrine signaling pathways (55, 56). Recent study data from patients with type 2 diabetes mellitus (T2DM) also suggest a dilating effect at the efferent arteriole with the same end effect of intraglomerular pressure reduction and decreasing hyperfiltration (57, 58). Reduction of intraglomerular pressure is a key factor for decreasing chronic loss of kidney function. In other words, in response to SGLT2 inhibition, less sodium and glucose are taken up in the proximal tubules by the SGLT2 and pass through the Henle loop, thus more sodium and glucose are seen by the macula densa with subsequently more adenosine generation, which increases afferent arteriole tone and decreases potentially efferent arteriole tone, hence reducing blood flow through the glomeruli and ultimately decreasing intraglomerular pressure (50, 51, 59). Decreasing intraglomerular pressure is one of the key pharmacological targets to protect the kidneys. Renin-angiotensin-aldosterone system (RAAS) blockers likewise protect the kidneys by decreasing intraglomerular pressure (60–63). Sensing the concentration of sodium and chloride ions in the lumen of the distal tubule via the Na^+^/K^+^-ATPase is an active process with ATP consumption. During ATP depletion, adenosine is formed. Adenosine binds to adenosine receptors (G-protein-coupled receptor) on the vascular smooth muscle cells of the afferent and efferent arteriole of the glomeruli thus reducing glomerular blood flow and intraglomerular pressure (64–66). Urinary adenosine excretion is a reliably indirect way to monitor drug effects on the TGF and was therefore used in our study (67–70). Our study was clearly able to link SGLT2 inhibitor-associated kidney protection in a classical nondiabetic CKD model to urinary adenosine excretion (Fig. 3). The correlation between Ccr, renal interstitial fibrosis, and glomerular size at study end with the urinary adenosine to creatinine ratio showed for the first time the relation between functional and structural hallmarks of CKD and urinary adenosine excretion as a surrogate parameter of the activity of the TGF. To finally prove the impact of an altered TGF by SGLT2 blockade on CKD outcome parameters, the investigation of the effects of the SGLT2 blocker in animal models without a TGF such as adenosine-1 receptor knockout mice would be helpful. Our current findings concerning TGF are in contrast to an earlier study of our group using also the 5/6 nephrectomy animal model and the same technical tools to investigate the empagliflozin effects. The only but key difference was the salt content of the food. In the previous study we gave a high-salt diet, whereas now we used the standard low-salt diet for rats. Our previous study showed no effect of empagliflozin on the urinary adenosine excretion in 5/6 nephrectomized rats on high-salt diet (29). The TGF mechanism as a target of SGLT2 blockade seems to be salt dependent—under high nutritional salt conditions, empagliflozin-mediated nephroprotective effects appeared to be TGF independent, whereas under normal salt intake, the TGF seems to play a key role (Fig. 3).

Other studies (39, 40, 43, 71–73), however, including our own recently published study (33), suggest that SGLT2 inhibition additionally ameliorates renal fibrosis in CKD rats by limiting macrophage differentiation and reducing infiltration of inflammatory cells and local expression of inflammatory factors. However, findings are controversial possibly partially due to the applied methods of investigating empagliflozin effects on inflammation. In our previous study, for example, we just applied single-cell RNA sequencing of the kidneys in a subset of animals due to budget restrictions without confirmation of findings by a second independent technology (33). We therefore now used a more sensitive method, immunofluorescence staining in all animals, to detect the number of CD4^+^ and CD8^+^ cells, as well as the relative fluorescence unit (RFU) of CD68^+^ cells in kidney tissues. There was no statistical difference in CD4^+^ and CD8^+^ T cells as well as CD68^+^ cells between groups (Fig. 4). Our current data are in agreement with a study analyzing the effects of 8 wk treatment with another SGLT2 blocker dapagliflozin of a nondiabetic CKD rat model (73). The reasons for overall controversial findings might be at least partially due to the methods used to detect inflammation, the CKD models used and timing of investigation after kidney injury as well as treatment duration with the SGLT2 blocker. Most of the previous studies were performed in diabetic models, whereas immunohistochemistry was more commonly used in nondiabetic models (40, 43, 72). In addition, in our current study treatment duration was 95 days, which is longer than in most other experiments. Inflammation might play no major pathophysiological role at this late stage anymore. Recently just differences in the expression of subtypes of macrophages were seen, an overall analysis of the density of macrophages might be a too crude tool to detect this (33). Given the complexity of findings reported concerning the mechanism of SGLT2 inhibition, we used additionally an open—nonhypothesis-driven approach—i.e., RNA sequencing followed by qRT-PCR to confirm findings from RNA sequencing. This strategy showed that components of the complement system—*C1QA* and *C1QC* mRNAs—were upregulated after 5/6 nephrectomy and this was corrected by empagliflozin in a dose-dependent manner (Fig. 5, *B* and *C*). *C1QA* and *C1QC* belong to *C1Q* (complement component 1Q subcomponent), the first component of complement that plays a crucial role in innate and adaptive immune responses (74). The activation of the complement system and the interaction between its components lead to a complement cascade reaction. Upregulated *C1Q* promotes the formation of its downstream C3 convertase (75) and stimulates the release of interleukin-12 (IL-12) (76), which ultimately produces the anaphylatoxins C3a and C5a via a downstream complement cascade reaction and the formation of the membrane attack complex. C3a and C5a play an important role on mediating the inflammatory and fibrotic process in the kidney (77–80). Some studies have already shown that *C1Q*-mediated increased synthesis of cytokines could further contribute to fibrosis by amplifying the interstitial inflammatory environment (81, 82). Interference with these mechanisms might likewise contribute to the beneficial effects of empagliflozin on CKD progression.

### Limitations

There are still some limitations to our experiment. First of all, the 5/6 nephrectomy model is an advanced model of chronic kidney disease, and therefore the results may not be generalizable to early stages of chronic kidney disease. Second, the effects of oral drugs are systemic, so there may be other mechanisms to improve renal function beyond the kidney, such as improvements in cardiac function and effects on metabolism, but we have only investigated the structure and function of the kidney. Furthermore, some clinical trials have shown changes in GFR over time, so that TGF may reset over time in response to SGLT2 inhibition, but we only measured urinary adenosine concentrations in rats at the end of the experiment. In addition, GFR was not directly measured, but it was roughly estimated by creatinine clearance calculated from urinary and blood concentrations of creatinine. Our experiments suggest two mechanisms of renoprotection by empagliflozin—TGF-mediated effects and alterations of gene expression of key components of the complement system; however, we did not explore the relationship between TGF and the complement system in further experiments, for example by blocking the TGF or analyzing the effects of empagliflozin in animal models without an intact TGF to observe potential TGF-related changes in the complement system. Moreover, the observed alterations in the mRNA expression of *C1QA* and *C1QC* genes could also be a secondary treatment effect, means secondary to primary effects of empagliflozin on for example renal fibrosis. Finally, we primarily focused on the pure effect of the treatment with an SGLT2 blocker in this study. But the combination of an SGLT2 blocker with an angiotensin receptor blocker would be of pharmacological as well as pathophysiological interest as well.

### Conclusions

Our experiments demonstrate that the nephroprotective effects of empagliflozin are not limited to diabetic nephropathy, but also improve the structure and function of nondiabetic nephropathy, including lowering serum creatinine, reducing proteinuria, increasing Ccr, and improving renal interstitial inflammation, renal interstitial fibrosis, perivascular fibrosis, and glomerulosclerosis. The nephroprotective effect of empagliflozin was more pronounced at higher doses (15 mg/kg/day) suggesting that this effect may be dose-dependent. Furthermore, the effects on urinary adenosine excretion suggest likewise a dose dependency of the effects of empagliflozin on the TGF. This suggests that the TGF-mediated effects on normalization of the glomerular pressure/glomerular hemodynamics might contribute to the nephroprotective effects of empagliflozin in a dose-dependent manner. Beyond the TGF effects, whole genome RNA sequencing followed by qRT-PCR of differently expressed genes in the kidneys suggest that empagliflozin limits complement cascade-mediated renal tissue damage in nondiabetic CKD by downregulating the expression of complement components *C1QA* and *C1QC* thus contributing to the SGLT2 blocker related amelioration of CKD progression.

## DATA AVAILABILITY

The data supporting the findings of this study are available in an online database. Raw data has been stored in figshare (raw data: https://doi.org/10.6084/m9.figshare.21587529.v1; Supplemental Figs. S1, S2, and S3: https://doi.org/10.6084/m9.figshare.21997511.v1).

## SUPPLEMENTAL DATA

10.6084/m9.figshare.21997511.v1 Supplemental Figs. S1–S3: https://doi.org/10.6084/m9.figshare.21997511.v1.

## GRANTS

China Scholarship Council supported X.C. and Y.C.

## DISCLOSURES

B.K.K. reports lecture fees and/or advisory board memberships and/or study participation from Astellas, Bayer, Boehringer Ingelheim, Chiesi, Riepharm, Pfizer, Sanofi, Servier and Vifor Pharma, all not related to the submitted work. D.D. and T.K. are research employees of Boehringer Ingelheim. None of the other authors has any conflicts of interest, financial or otherwise, to disclose. 

## AUTHOR CONTRIBUTIONS

B. Hocher conceived and designed research; X.C., Y.C., Z.Z., H.W., and L.Y. performed experiments; X.C., D.D., Y.C., L.S., Q.S., H.W., A.A.H., M.M.S.G., B.K.K., T.K., L.Y., B. He and B. Hocher analyzed data; X.C., D.D., Y.C., L.S., Q.S., A.A.H., B.K.K., T.K., and B. Hocher interpreted results of experiments; X.C., M.M.S.G., and B.K.K. prepared figures; X.C. and B. Hocher drafted manuscript; X.C., D.D., A.A.H., C.R., B.K.K., T.K., B. He, S.M., and B. Hocher edited and revised manuscript; X.C., D.D., Y.C., L.S., Q.S., Z.Z., A.A.H., B.K.K., T.K., B. He, S.M., and B. Hocher approved final version of manuscript.

## References

[1] Stevens PE, Levin A; Kidney Disease: Improving Global Outcomes Chronic Kidney Disease Guideline Development Work Group Members. Evaluation and management of chronic kidney disease: synopsis of the kidney disease: improving global outcomes 2012 clinical practice guideline. Ann Intern Med 158: 825–830, 2013. doi:10.7326/0003-4819-158-11-201306040-00007. 23732715

[2] Webster AC, Nagler EV, Morton RL, Masson P. Chronic kidney disease. Lancet 389: 1238–1252, 2017. doi:10.1016/S0140-6736(16)32064-5. 27887750

[3] Drawz P, Rahman M. Chronic kidney disease. Ann Intern Med 162: ITC1–ITC16, 2015. doi:10.7326/AITC201506020.26030647

[4] Rayego-Mateos S, Valdivielso JM. New therapeutic targets in chronic kidney disease progression and renal fibrosis. Expert Opin Ther Targets 24: 655–670, 2020. doi:10.1080/14728222.2020.1762173. 32338087

[5] Nespoux J, Vallon V. SGLT2 inhibition and kidney protection. Clin Sci (Lond) 132: 1329–1339, 2018. doi:10.1042/CS20171298. 29954951 PMC6648703

[6] Neal B, Perkovic V, Mahaffey KW, de Zeeuw D, Fulcher G, Erondu N, Shaw W, Law G, Desai M, Matthews DR, Group CPC; CANVAS Program Collaborative Group. Canagliflozin and cardiovascular and renal events in type 2 diabetes. N Engl J Med 377: 644–657, 2017. doi:10.1056/NEJMoa1611925. 28605608

[7] Wiviott SD, Raz I, Bonaca MP, Mosenzon O, Kato ET, Cahn A, Silverman MG, Zelniker TA, Kuder JF, Murphy SA, Bhatt DL, Leiter LA, McGuire DK, Wilding JPH, Ruff CT, Gause-Nilsson IAM, Fredriksson M, Johansson PA, Langkilde AM, Sabatine MS; DECLARE-TIMI 58 Investigators. Dapagliflozin and cardiovascular outcomes in type 2 diabetes. N Engl J Med 380: 347–357, 2019. doi:10.1056/NEJMoa1812389. 30415602

[8] Zinman B, Wanner C, Lachin JM, Fitchett D, Bluhmki E, Hantel S, Mattheus M, Devins T, Johansen OE, Woerle HJ, Broedl UC, Inzucchi SE; EMPA-REG OUTCOME Investigators. Empagliflozin, cardiovascular outcomes, and mortality in type 2 diabetes. N Engl J Med 373: 2117–2128, 2015. doi:10.1056/NEJMoa1504720. 26378978

[9] Wanner C, Inzucchi SE, Lachin JM, Fitchett D, von Eynatten M, Mattheus M, Johansen OE, Woerle HJ, Broedl UC, Zinman B; EMPA-REG OUTCOME Investigators. Empagliflozin and progression of kidney disease in type 2 diabetes. N Engl J Med 375: 323–334, 2016. doi:10.1056/NEJMoa1515920. 27299675

[10] Heerspink HJL, Stefansson BV, Correa-Rotter R, Chertow GM, Greene T, Hou FF, Mann JFE, McMurray JJV, Lindberg M, Rossing P, Sjostrom CD, Toto RD, Langkilde AM, Wheeler DC; DAPA-CKD Trial Committees and Investigators. Dapagliflozin in patients with chronic kidney disease. N Engl J Med 383: 1436–1446, 2020. doi:10.1056/NEJMoa2024816. 32970396

[11] Herrington WG, Staplin N, Wanner C, Green JB, Hauske SJ, Emberson JR, et al; The EMPA-KIDNEY Collaborative Group. Empagliflozin in patients with chronic kidney disease. N Engl J Med 388: 117–127, 2022. doi:10.1056/NEJMoa2204233. 36331190 PMC7614055

[12] Packer M. Interplay of adenosine monophosphate-activated protein kinase/sirtuin-1 activation and sodium influx inhibition mediates the renal benefits of sodium-glucose co-transporter-2 inhibitors in type 2 diabetes: a novel conceptual framework. Diabetes Obes Metab 22: 734–742, 2020. doi:10.1111/dom.13961. 31916329

[13] Packer M. Mechanisms leading to differential hypoxia-inducible factor signaling in the diabetic kidney: modulation by SGLT2 inhibitors and hypoxia mimetics. Am J Kidney Dis 77: 280–286, 2021. doi:10.1053/j.ajkd.2020.04.016. 32711072

[14] Mima A. Sodium-glucose cotransporter 2 inhibitors in patients with non-diabetic chronic kidney disease. Adv Ther 38: 2201–2212, 2021. doi:10.1007/s12325-021-01735-5. 33860925

[15] Thomson SC, Vallon V. Effects of SGLT2 inhibitor and dietary NaCl on glomerular hemodynamics assessed by micropuncture in diabetic rats. Am J Physiol Renal Physiol 320: F761–F771, 2021. doi:10.1152/ajprenal.00552.2020. 33645318 PMC8174804

[16] Thomas MC, Cherney DZI. The actions of SGLT2 inhibitors on metabolism, renal function and blood pressure. Diabetologia 61: 2098–2107, 2018. doi:10.1007/s00125-018-4669-0. 30132034

[17] Szekeres Z, Toth K, Szabados E. The effects of SGLT2 inhibitors on lipid metabolism. Metabolites 11: 87, 2021. doi:10.3390/metabo11020087. 33535652 PMC7912792

[18] Ruggenenti P, Perna A, Gherardi G, Garini G, Zoccali C, Salvadori M, Scolari F, Schena FP, Remuzzi G. Renoprotective properties of ACE-inhibition in non-diabetic nephropathies with non-nephrotic proteinuria. Lancet 354: 359–364, 1999. doi:10.1016/S0140-6736(98)10363-X. 10437863

[19] Hou FF, Zhang X, Zhang GH, Xie D, Chen PY, Zhang WR, Jiang JP, Liang M, Wang GB, Liu ZR, Geng RW. Efficacy and safety of benazepril for advanced chronic renal insufficiency. N Engl J Med 354: 131–140, 2006. doi:10.1056/NEJMoa053107. 16407508

[20] Brenner BM, Cooper ME, de Zeeuw D, Keane WF, Mitch WE, Parving HH, Remuzzi G, Snapinn SM, Zhang Z, Shahinfar S; RENAAL Study Investigators. Effects of losartan on renal and cardiovascular outcomes in patients with type 2 diabetes and nephropathy. N Engl J Med 345: 861–869, 2001. doi:10.1056/NEJMoa011161. 11565518

[21] Lewis EJ, Hunsicker LG, Clarke WR, Berl T, Pohl MA, Lewis JB, Ritz E, Atkins RC, Rohde R, Raz I; Collaborative Study Group. Renoprotective effect of the angiotensin-receptor antagonist irbesartan in patients with nephropathy due to type 2 diabetes. N Engl J Med 345: 851–860, 2001. doi:10.1056/NEJMoa011303. 11565517

[22] Tsuprykov O, Ando R, Reichetzeder C, von Websky K, Antonenko V, Sharkovska Y, Chaykovska L, Rahnenfuhrer J, Hasan AA, Tammen H, Alter M, Klein T, Ueda S, Yamagishi SI, Okuda S, Hocher B. The dipeptidyl peptidase inhibitor linagliptin and the angiotensin II receptor blocker telmisartan show renal benefit by different pathways in rats with 5/6 nephrectomy. Kidney Int 89: 1049–1061, 2016. doi:10.1016/j.kint.2016.01.016. 27083282

[23] Delic D, Wiech F, Urquhart R, Gabrielyan O, Rieber K, Rolser M, Tsuprykov O, Hasan AA, Kramer BK, Baum P, Kohler A, Gantner F, Mark M, Hocher B, Klein T. Linagliptin and telmisartan induced effects on renal and urinary exosomal miRNA expression in rats with 5/6 nephrectomy. Sci Rep 10: 3373, 2020. doi:10.1038/s41598-020-60336-4. 32099009 PMC7042229

[24] Zeng S, Delic D, Chu C, Xiong Y, Luo T, Chen X, Gaballa MMS, Xue Y, Chen X, Cao Y, Hasan AA, Stadermann K, Frankenreiter S, Yin L, Kramer BK, Klein T, Hocher B. Antifibrotic effects of low dose SGLT2 Inhibition with empagliflozin in comparison to Ang II receptor blockade with telmisartan in 5/6 nephrectomised rats on high salt diet. Biomed Pharmacother 146: 112606, 2022. doi:10.1016/j.biopha.2021.112606. 34968924

[25] Sarashina A, Koiwai K, Seman LJ, Yamamura N, Taniguchi A, Negishi T, Sesoko S, Woerle HJ, Dugi KA. Safety, tolerability, pharmacokinetics and pharmacodynamics of single doses of empagliflozin, a sodium glucose cotransporter 2 (SGLT2) inhibitor, in healthy Japanese subjects. Drug Metab Pharmacokinet 28: 213–219, 2013. doi:10.2133/dmpk.dmpk-12-rg-082. 23149871

[26] Grempler R, Thomas L, Eckhardt M, Himmelsbach F, Sauer A, Sharp DE, Bakker RA, Mark M, Klein T, Eickelmann P. Empagliflozin, a novel selective sodium glucose cotransporter-2 (SGLT-2) inhibitor: characterisation and comparison with other SGLT-2 inhibitors. Diabetes Obes Metab 14: 83–90, 2012. doi:10.1111/j.1463-1326.2011.01517.x. 21985634

[27] Rajasekeran H, Lytvyn Y, Bozovic A, Lovshin JA, Diamandis E, Cattran D, Husain M, Perkins BA, Advani A, Reich HN, Kulasingam V, Cherney DZI. Urinary adenosine excretion in type 1 diabetes. Am J Physiol Renal Physiol 313: F184–F191, 2017. doi:10.1152/ajprenal.00043.2017. 28381459

[28] Dias BCL, Fachi MM, de Campos ML, Degaut FLD, Peccinini RG, Pontarolo R. A new HPLC-MS/MS method for the simultaneous quantification of SGLT2 inhibitors and metformin in plasma and its application to a pharmacokinetic study in healthy volunteers. Biomed Chromatogr 33: e4663, 2019. doi:10.1002/bmc.4663. 31339572

[29] Zeng S, Delic D, Chu C, Xiong Y, Luo T, Chen X, Gaballa MMS, Xue Y, Chen X, Cao Y, Hasan AA, Stadermann K, Frankenreiter S, Yin L, Kramer BK, Klein T, Hocher B. Antifibrotic effects of low dose SGLT2 Inhibition with empagliflozin in comparison to Ang II receptor blockade with telmisartan in 5/6 nephrectomised rats on high salt diet. Biomed Pharmacother 146: 112606, 2022. doi:10.1016/j.biopha.2021.112606. 34968924

[30] Kanasaki K, Shi S, Kanasaki M, He J, Nagai T, Nakamura Y, Ishigaki Y, Kitada M, Srivastava SP, Koya D. Linagliptin-mediated DPP-4 inhibition ameliorates kidney fibrosis in streptozotocin-induced diabetic mice by inhibiting endothelial-to-mesenchymal transition in a therapeutic regimen. Diabetes 63: 2120–2131, 2014. doi:10.2337/db13-1029. 24574044

[31] Winiarska A, Knysak M, Nabrdalik K, Gumprecht J, Stompor T. Inflammation and oxidative stress in diabetic kidney disease: the targets for SGLT2 inhibitors and GLP-1 receptor agonists. Int J Mol Sci 22: 10822, 2021. doi:10.3390/ijms221910822. 34639160 PMC8509708

[32] DeFronzo RA, Reeves WB, Awad AS. Pathophysiology of diabetic kidney disease: impact of SGLT2 inhibitors. Nat Rev Nephrol 17: 319–334, 2021. doi:10.1038/s41581-021-00393-8. 33547417

[33] Lu YP, Wu HW, Zhu T, Li XT, Zuo J, Hasan AA, Reichetzeder C, Delic D, Yard B, Klein T, Kramer BK, Zhang ZY, Wang XH, Yin LH, Dai Y, Zheng ZH, Hocher B. Empagliflozin reduces kidney fibrosis and improves kidney function by alternative macrophage activation in rats with 5/6-nephrectomy. Biomed Pharmacother 156: 113947, 2022. doi:10.1016/j.biopha.2022.113947. 36411661

[34] Wagner J, Drab M, Bohlender J, Amann K, Wienen W, Ganten D. Effects of AT1 receptor blockade on blood pressure and the renin-angiotensin system in spontaneously hypertensive rats of the stroke prone strain. Clin Exp Hypertens 20: 205–221, 1998. doi:10.3109/10641969809053215. 9533614

[35] Te Riet L, van Esch JH, Roks AJ, van den Meiracker AH, Danser AH. Hypertension: renin-angiotensin-aldosterone system alterations. Circ Res 116: 960–975, 2015. doi:10.1161/CIRCRESAHA.116.303587. 25767283

[36] Ahmed AS, Mona MM, Abdel-Kareem MA, Elsisy RA. SGLT2 inhibitor empagliflozin monotherapy alleviates renal oxidative stress in albino Wistar diabetic rats after myocardial infarction induction. Biomed Pharmacother 139: 111624, 2021. doi:10.1016/j.biopha.2021.111624. 33915503

[37] Abdel-Wahab AF, Bamagous GA, Al-Harizy RM, ElSawy NA, Shahzad N, Ibrahim IA, Ghamdi SSA. Renal protective effect of SGLT2 inhibitor dapagliflozin alone and in combination with irbesartan in a rat model of diabetic nephropathy. Biomed Pharmacother 103: 59–66, 2018. doi:10.1016/j.biopha.2018.03.176. 29635129

[38] Domon A, Katayama K, Sato T, Tochigi Y, Tazaki H, Suzuki H. Empagliflozin ameliorates symptoms of diabetes and renal tubular dysfunction in a rat model of diabetes with enlarged kidney (DEK). PLoS One 16: e0251135, 2021. doi:10.1371/journal.pone.0251135. 33945582 PMC8096081

[39] Cassis P, Locatelli M, Cerullo D, Corna D, Buelli S, Zanchi C, Villa S, Morigi M, Remuzzi G, Benigni A, Zoja C. SGLT2 inhibitor dapagliflozin limits podocyte damage in proteinuric nondiabetic nephropathy. JCI Insight 3: e98720, 2018. doi:10.1172/jci.insight.98720. 30089717 PMC6129124

[40] Castoldi G, Carletti R, Ippolito S, Colzani M, Barzaghi F, Stella A, Zerbini G, Perseghin G, Zatti G, di Gioia CRT. Sodium-glucose cotransporter 2 inhibition prevents renal fibrosis in cyclosporine nephropathy. Acta Diabetol 58: 1059–1070, 2021. doi:10.1007/s00592-021-01681-2. 33760995 PMC8272713

[41] Yamato M, Kato N, Kakino A, Yamada KI, Inoguchi T. Low dose of sodium-glucose transporter 2 inhibitor ipragliflozin attenuated renal dysfunction and interstitial fibrosis in adenine-induced chronic kidney disease in mice without diabetes. Metabol Open 7: 100049, 2020. doi:10.1016/j.metop.2020.100049. 33015603 PMC7520892

[42] Abbas NAT, El Salem A, Awad MM. Empagliflozin, SGLT2 inhibitor, attenuates renal fibrosis in rats exposed to unilateral ureteric obstruction: potential role of klotho expression. Naunyn Schmiedebergs Arch Pharmacol 391: 1347–1360, 2018. doi:10.1007/s00210-018-1544-y. 30090949

[43] Kim S, Jo CH, Kim GH. Effects of empagliflozin on nondiabetic salt-sensitive hypertension in uninephrectomized rats. Hypertens Res 42: 1905–1915, 2019. doi:10.1038/s41440-019-0326-3. 31537914 PMC8075936

[44] Li L, Konishi Y, Morikawa T, Zhang Y, Kitabayashi C, Kobara H, Masaki T, Nakano D, Hitomi H, Kobori H, Nishiyama A. Effect of a SGLT2 inhibitor on the systemic and intrarenal renin-angiotensin system in subtotally nephrectomized rats. J Pharmacol Sci 137: 220–223, 2018. doi:10.1016/j.jphs.2017.10.006. 29983235 PMC6050139

[45] Zhang Y, Thai K, Kepecs DM, Gilbert RE. Sodium-glucose linked cotransporter-2 inhibition does not attenuate disease progression in the rat remnant kidney model of chronic kidney disease. PLoS One 11: e0144640, 2016. doi:10.1371/journal.pone.0144640. 26741142 PMC4711803

[46] Ku EJ, Lee DH, Jeon HJ, Oh TK. Empagliflozin versus dapagliflozin in patients with type 2 diabetes inadequately controlled with metformin, glimepiride and dipeptidyl peptide 4 inhibitors: a 52-week prospective observational study. Diabetes Res Clin Pract 151: 65–73, 2019. doi:10.1016/j.diabres.2019.04.008. 30954510

[47] Ku EJ, Lee DH, Jeon HJ, Oh TK. Long-term effectiveness and safety of quadruple combination therapy with empagliflozin versus dapagliflozin in patients with type 2 diabetes: 3-year prospective observational study. Diabetes Res Clin Pract 182: 109123, 2021. doi:10.1016/j.diabres.2021.109123. 34740742

[48] Tauber P, Sinha F, Berger RS, Gronwald W, Dettmer K, Kuhn M, Trum M, Maier LS, Wagner S, Schweda F. Empagliflozin reduces renal hyperfiltration in response to uninephrectomy, but is not nephroprotective in UNx/DOCA/salt mouse models. Front Pharmacol 12: 761855, 2021. doi:10.3389/fphar.2021.761855. 34992532 PMC8724563

[49] Corremans R, Neven E, Maudsley S, Leysen H, De Broe ME, D'Haese PC, Vervaet BA, Verhulst A. Progression of established non-diabetic chronic kidney disease is halted by metformin treatment in rats. Kidney Int 101: 929–944, 2022. doi:10.1016/j.kint.2022.01.037. 35271933

[50] Perrone-Filardi P, Avogaro A, Bonora E, Colivicchi F, Fioretto P, Maggioni AP, Sesti G, Ferrannini E. Mechanisms linking empagliflozin to cardiovascular and renal protection. Int J Cardiol 241: 450–456, 2017. doi:10.1016/j.ijcard.2017.03.089. 28395981

[51] Cherney DZ, Perkins BA, Soleymanlou N, Maione M, Lai V, Lee A, Fagan NM, Woerle HJ, Johansen OE, Broedl UC, von Eynatten M. Renal hemodynamic effect of sodium-glucose cotransporter 2 inhibition in patients with type 1 diabetes mellitus. Circulation 129: 587–597, 2014. doi:10.1161/CIRCULATIONAHA.113.005081. 24334175

[52] Thurau K, Schnermann J. The sodium concentration in the macula densa cells as a regulating factor for glomerular filtration (micropuncture experiments). Klin Wochenschr 43: 410–413, 1965. doi:10.1007/BF01483845. 14333330

[53] Oyarzun C, Garrido W, Alarcon S, Yanez A, Sobrevia L, Quezada C, San MR. Adenosine contribution to normal renal physiology and chronic kidney disease. Mol Aspects Med 55: 75–89, 2017. doi:10.1016/j.mam.2017.01.004. 28109856

[54] Zehra T, Cupples WA, Braam B. Tubuloglomerular feedback synchronization in nephrovascular networks. J Am Soc Nephrol 32: 1293–1304, 2021. doi:10.1681/ASN.2020040423. 33833078 PMC8259654

[55] Fioretto P, Zambon A, Rossato M, Busetto L, Vettor R. SGLT2 inhibitors and the diabetic kidney. Diabetes Care 39, Suppl 2: S165–171, 2016. doi:10.2337/dcS15-3006. 27440829

[56] Vallon V, Gerasimova M, Rose MA, Masuda T, Satriano J, Mayoux E, Koepsell H, Thomson SC, Rieg T. SGLT2 inhibitor empagliflozin reduces renal growth and albuminuria in proportion to hyperglycemia and prevents glomerular hyperfiltration in diabetic Akita mice. Am J Physiol Renal Physiol 306: F194–F204, 2014. doi:10.1152/ajprenal.00520.2013. 24226524 PMC3920018

[57] Nordquist L, Brown R, Fasching A, Persson P, Palm F. Proinsulin C-peptide reduces diabetes-induced glomerular hyperfiltration via efferent arteriole dilation and inhibition of tubular sodium reabsorption. Am J Physiol Renal Physiol 297: F1265–F1272, 2009. doi:10.1152/ajprenal.00228.2009. 19741019 PMC2781335

[58] Persson P, Hansell P, Palm F. Reduced adenosine A2a receptor-mediated efferent arteriolar vasodilation contributes to diabetes-induced glomerular hyperfiltration. Kidney Int 87: 109–115, 2015. doi:10.1038/ki.2014.219. 24940797

[59] Skrtic M, Cherney DZ. Sodium-glucose cotransporter-2 inhibition and the potential for renal protection in diabetic nephropathy. Curr Opin Nephrol Hypertens 24: 96–103, 2015. doi:10.1097/MNH.0000000000000084. 25470017

[60] Kobori H, Mori H, Masaki T, Nishiyama A. Angiotensin II blockade and renal protection. Curr Pharm Des 19: 3033–3042, 2013. doi:10.2174/1381612811319170009. 23176216 PMC3651580

[61] Barnett AH. Preventing renal complications in diabetic patients: the Diabetics Exposed to Telmisartan And enalaprIL (DETAIL) study. Acta Diabetol 42, *Suppl*1: S42–S49, 2005. doi:10.1007/s00592-005-0180-4. 15868119

[62] Kobori H, Nangaku M, Navar LG, Nishiyama A. The intrarenal renin-angiotensin system: from physiology to the pathobiology of hypertension and kidney disease. Pharmacol Rev 59: 251–287, 2007. doi:10.1124/pr.59.3.3. 17878513

[63] Imanishi M, Yoshioka K, Konishi Y, Okumura M, Okada N, Sato T, Tanaka S, Fujii S, Kimura G. Glomerular hypertension as one cause of albuminuria in type II diabetic patients. Diabetologia 42: 999–1005, 1999. doi:10.1007/s001250051259. 10491761

[64] Castrop H. Mediators of tubuloglomerular feedback regulation of glomerular filtration: ATP and adenosine. Acta Physiol (Oxf) 189: 3–14, 2007. doi:10.1111/j.1748-1716.2006.01610.x. 17280552

[65] Osswald H, Muhlbauer B, Vallon V. Adenosine and tubuloglomerular feedback. Blood Purif 15: 243–252, 1997. doi:10.1159/000170342. 9435952

[66] Carlstrom M, Wilcox CS, Welch WJ. Adenosine A(2) receptors modulate tubuloglomerular feedback. Am J Physiol Renal Physiol 299: F412–F417, 2010. doi:10.1152/ajprenal.00211.2010. 20519378 PMC2928527

[67] Persson AE, Lai EY, Gao X, Carlstrom M, Patzak A. Interactions between adenosine, angiotensin II and nitric oxide on the afferent arteriole influence sensitivity of the tubuloglomerular feedback. Front Physiol 4: 187, 2013. doi:10.3389/fphys.2013.00187. 23882224 PMC3714451

[68] Wanner C. Sodium glucose cotransporter 2 inhibition and the visualization of kidney hemodynamics. Circulation 140: 316–318, 2019. doi:10.1161/CIRCULATIONAHA.119.040326. 31329485

[69] Marton A, Kaneko T, Kovalik JP, Yasui A, Nishiyama A, Kitada K, Titze J. Organ protection by SGLT2 inhibitors: role of metabolic energy and water conservation. Nat Rev Nephrol 17: 65–77, 2021. doi:10.1038/s41581-020-00350-x. 33005037

[70] Kidokoro K, Cherney DZI, Bozovic A, Nagasu H, Satoh M, Kanda E, Sasaki T, Kashihara N. Evaluation of glomerular hemodynamic function by empagliflozin in diabetic mice using in vivo imaging. Circulation 140: 303–315, 2019. doi:10.1161/CIRCULATIONAHA.118.037418. 30773020

[71] Vallon V, Verma S. Effects of SGLT2 inhibitors on kidney and cardiovascular function. Annu Rev Physiol 83: 503–528, 2021. doi:10.1146/annurev-physiol-031620-095920. 33197224 PMC8017904

[72] Castoldi G, Carletti R, Ippolito S, Colzani M, Barzaghi F, Stella A, Zerbini G, Perseghin G, di Gioia CRT. Renal anti-fibrotic effect of sodium glucose cotransporter 2 inhibition in angiotensin II-dependent hypertension. Am J Nephrol 51: 119–129, 2020. doi:10.1159/000505144. 31910407

[73] Rajasekeran H, Reich HN, Hladunewich MA, Cattran D, Lovshin JA, Lytvyn Y, Bjornstad P, Lai V, Tse J, Cham L, Majumder S, Bowskill BB, Kabir MG, Advani SL, Gibson IW, Sood MM, Advani A, Cherney DZI. Dapagliflozin in focal segmental glomerulosclerosis: a combined human-rodent pilot study. Am J Physiol Renal Physiol 314: F412–F422, 2018. doi:10.1152/ajprenal.00445.2017. 29141939 PMC5899226

[74] Kishore U, Reid KB. C1q: structure, function, and receptors. Immunopharmacology 49: 159–170, 2000. doi:10.1016/s0162-3109(00)80301-x. 10904115

[75] Muller-Eberhard HJ. Molecular organization and function of the complement system. Annu Rev Biochem 57: 321–347, 1988. doi:10.1146/annurev.bi.57.070188.001541. 3052276

[76] Tang S, Sheerin NS, Zhou W, Brown Z, Sacks SH. Apical proteins stimulate complement synthesis by cultured human proximal tubular epithelial cells. J Am Soc Nephrol 10: 69–76, 1999. doi:10.1681/ASN.V10169. 9890311

[77] Peng Q, Li K, Patel H, Sacks SH, Zhou W. Dendritic cell synthesis of C3 is required for full T cell activation and development of a Th1 phenotype. J Immunol 176: 3330–3341, 2006. doi:10.4049/jimmunol.176.6.3330. 16517700

[78] Li K, Fazekasova H, Wang N, Peng Q, Sacks SH, Lombardi G, Zhou W. Functional modulation of human monocytes derived DCs by anaphylatoxins C3a and C5a. Immunobiology 217: 65–73, 2012. doi:10.1016/j.imbio.2011.07.033. 21855168 PMC3234345

[79] Bora PS, Sohn JH, Cruz JM, Jha P, Nishihori H, Wang Y, Kaliappan S, Kaplan HJ, Bora NS. Role of complement and complement membrane attack complex in laser-induced choroidal neovascularization. J Immunol 174: 491–497, 2005. doi:10.4049/jimmunol.174.1.491. 15611275

[80] Huang G, Zhang Y, Kim B, Ge G, Annis DS, Mosher DF, Greenspan DS. Fibronectin binds and enhances the activity of bone morphogenetic protein 1. J Biol Chem 284: 25879–25888, 2009. doi:10.1074/jbc.M109.024125. 19617627 PMC2757989

[81] Xavier S, Sahu RK, Landes SG, Yu J, Taylor RP, Ayyadevara S, Megyesi J, Stallcup WB, Duffield JS, Reis ES, Lambris JD, Portilla D. Pericytes and immune cells contribute to complement activation in tubulointerstitial fibrosis. Am J Physiol Renal Physiol 312: F516–F532, 2017. doi:10.1152/ajprenal.00604.2016. 28052876 PMC5374314

[82] Portilla D, Xavier S. Role of intracellular complement activation in kidney fibrosis. Br J Pharmacol 178: 2880–2891, 2021. doi:10.1111/bph.15408. 33555070

